# MerR-type regulator ZccR links zinc homeostasis to biofilm formation and interspecies competition in *Streptococcus mutans*

**DOI:** 10.3389/fcimb.2026.1946820

**Published:** 2026-09-30

**Authors:** Yangyang Pan, Zhaonan Xu, Shuyu Wang, Muxin Xu, Haiyun Dong, Liqin Mei, Keke Zhang

**Affiliations:** 1 Department of Pediatric Dentistry, School and Hospital of Stomatology, Wenzhou Medical University, Wenzhou, China; 2 Institute of Stomatology, School and Hospital of Stomatology, Wenzhou Medical University, Wenzhou, China

**Keywords:** biofilm formation, interspecies competition, oral microecology, *Streptococcus mutans*, transcriptional regulation, ZccR, zinc homeostasis

## Abstract

**Objectives:**

Zinc is widely present in the oral microenvironment and can influence the growth of oral bacteria. ZccR is a MerR-type transcriptional regulator that controls zinc efflux in *Streptococcus mutans* (*S. mutans*), but its role in biofilm virulence and interspecies competition with oral commensals remains unclear. This study aimed to investigate the role of ZccR in the formation of single-species biofilms of *S. mutans* and dual-species biofilms with oral commensal streptococci, and its underlying regulatory mechanisms.

**Methods:**

We constructed an in-frame *zccR* knockout mutant of *S. mutans*. The growth curve, crystal violet staining, anthrone method, scanning electron microscopy, fluorescence *in situ* hybridization, and deferred antagonism assay were used to assess growth, biofilm development, dual-species biofilm composition, and interspecies competitive capacity. RNA-seq was employed to identify genome-wide transcriptional alterations, and promoter-*lacZ* reporter strains were constructed to confirm the transcriptional regulation mode of ZccR on *zccE* and on itself.

**Results:**

The deletion of *zccR* significantly suppressed biofilm formation and water-insoluble exopolysaccharide production of *S. mutans* at 150 μM Zn^2+^, a concentration that only modestly affected planktonic growth. In dual-species biofilms containing *Streptococcus gordonii (S. gordonii)*, the mutant exhibited reduced oxidative stress tolerance and weaker inhibition of *S. gordonii*, despite upregulation of bacteriocin genes. RNA-sequencing identified 89 differentially expressed genes (67 upregulated and 22 downregulated), including downregulation of the mutanobactin cluster and FAD-dependent oxidoreductases, upregulation of iron-uptake genes, induction of HomR–*tcy*-linked cystine-uptake pathways, glutamine biosynthesis, and CRISPR-Cas genes, and a shift from sucrose import (*scrA/msmX*) toward *lac*-dependent lactose/galactose metabolism. Most of these shifts tracked intracellular zinc accumulation rather than direct ZccR control. Promoter-*lacZ* assays revealed that ZccR activates *zccE* and represses its own promoter, and zinc acts on these promoters through ZccR-dependent and ZccR-independent routes.

**Conclusions:**

ZccR links zinc homeostasis with biofilm formation and oral interspecies ecology of *S. mutans*, thus offering a future intervention point for cariogenic biofilm control.

## 1 Introduction

Dental caries is the most prevalent oral disease globally, affecting individuals of all ages and backgrounds. Global statistics indicate that approximately 2.24 billion people are affected, with untreated dental caries in permanent teeth having an age-standardized prevalence of 27,500 per 100,000 individuals (GBD 2021 Oral Disorders Collaborators, 2025; Li et al., 2025). Significant increases in disease burden were observed from 1990 to 2021, especially in China and Colombia (Li et al., 2025). Consequently, the prevention and treatment of caries remain a significant concern.

Dental plaque is implicated in caries development. In a healthy condition, the microorganisms within dental plaque are in a state of dynamic equilibrium with intermicrobial competition, contributing substantially to the maintenance of microbial homeostasis. For instance, dynamic interactions between the conditional cariogenic *Streptococcus mutans* (*S. mutans*) and primary inhabitants *Streptococcus sanguinis* (*S. sanguinis*) and *Streptococcus gordonii* (*S. gordonii*) contribute to this microbial balance (Ye et al., 2025). As a principal type of cariogenic bacteria, *S. mutans* harbors essential virulence factors for the pathogenesis of cavities. It synthesizes glucans for biofilm formation and converts sugars into lactic acid, initiating enamel demineralization. Its resilience to acidic environments and ability to synthesize bacteriocins provide it with a competitive edge, allowing it to thrive in acidic milieu and prevail in shifting oral microenvironments (Zhang et al., 2021; He et al., 2022; Yu et al., 2023). Conversely, *S. sanguinis* and *S. gordonii* synthesize hydrogen peroxide (H_2_O_2_) and other antimicrobial agents as part of their competitive mechanisms to prevent the overgrowth of *S. mutans* (Huang et al., 2018). However, an imbalance in the oral microbiota, coupled with the ecological dominance of acid-producing and acid-tolerant bacteria, leads to dental caries development (Ye et al., 2025). Accordingly, strategies that target conditional cariogenic bacteria, including *S. mutans*, which subsequently affects the microbial composition within the plaque biofilm, have potential for effective caries prevention and management (Cao et al., 2025).

The oral cavity provides a unique, complex habitat for diverse microbial communities. Factors, including metal ions within the oral cavity, might influence the structure and function of the biofilm (Toyofuku et al., 2016; Li et al., 2022). Increased calcium ion supplementation increases the biomass and metabolic activity of saliva-derived biofilms and alters their microbial community composition, illustrating the influence of microenvironmental changes on oral microbial dynamics (Shokeen et al., 2022). Another notable example is the zinc ion (Zn^2+^). Zinc, an essential trace metal for living cells, is prevalent in saliva, dental plaque, and tooth enamel’s hydroxyapatite, serving as an essential environmental modulator in the oral cavity. Zinc is widely used in oral health care products, dental biomaterials, and orthodontic materials because of its multifunctional properties, including the prevention of dental calculus, enhancement of immunity, and impact on cell division and skeletal development (Makthal and Kumaraswami, 2017; Uwitonze et al., 2020). Zinc demonstrates prolonged retention at elevated concentrations, especially in dental plaque and saliva, after being introduced by foods, mouthwashes, and toothpaste (up to 500 μM or even higher). However, physiological zinc concentrations in oral fluids sustain a narrow equilibrium range (0.2–3 μM), suggesting that using high-concentration zinc products and foods might cause significant fluctuations in the oral zinc milieu (Lynch, 2011). Studies have demonstrated that environmental zinc levels significantly affect biofilm formation in various pathogenic species, including *Actinobacillus pleuropneumoniae, Salmonella typhimurium, Haemophilus parasuis, Escherichia coli*, and *Staphylococcus aureus* (Wu et al., 2013; Makthal and Kumaraswami, 2017). Consequently, changes in the oral zinc milieu may significantly alter the ecophysiology of oral biofilms.

However, previous studies have demonstrated that *S. mutans* exhibits significantly higher zinc tolerance (minimum inhibitory concentration [MIC] = 4 mM) compared to other species tested, including oral commensals (*S. gordonii, S. sanguinis*) and *pyogenic streptococci* (*S. agalactiae, S. pyogenes*), which have MIC values ranging from 0.5 to 1 mM (Ganguly et al., 2022). This enhanced zinc resistance in *S. mutans* is mostly attributed to its zinc homeostasis regulatory transcription factors. Under high-zinc stress, the zinc uptake regulator AdcR inhibits the expression of *adcA* and *adcBC* to prevent excessive Zn^2+^ absorption, whereas the zinc efflux-related transcription factor ZccR facilitates Zn^2+^ extrusion. Compared to the wild type, the AdcR knockout strain exhibited greater growth inhibition under severe zinc stress, accompanied by increased extracellular polysaccharide (EPS) production, whereas the loss of *adcBC* hindered growth and reduced utilization of various carbohydrates of *S. mutans* (Pedrosa et al., 2026; Pan et al., 2021). Previous observations indicate that deletion of ZccR led to an approximately 10-fold increase in intracellular zinc accumulation upon exposure to 1 mM Zn^2+^ (Ganguly et al., 2022). However, the relationship between zinc efflux regulator ZccR and biofilm development, especially regarding cariogenic biofilm architecture, remains poorly understood and warrants further investigation.

This study examined the impact of *zccR* gene knockout on *S. mutans* biofilm development and its effect on dual-species biofilms with oral commensal streptococci under zinc treatment (0–500 μM Zn^2+^, a physiologically attainable, sub-MIC zinc challenge that models the zinc spikes encountered by *S. mutans* in the oral microenvironment). Additionally, we examined the impact of this transcriptional regulator on the genome-wide transcription of *S. mutans* and its regulatory role in the transcriptional control of the ZccR-associated zinc efflux system.

## 2 Materials and methods

### 2.1 Bacterial strains and growth conditions

*S. mutans* UA159 ATCC 700610, *S. gordonii* DL1, *Streptococcus salivarius* ATCC 7073, and *Enterococcus faecalis* ATCC 29212 were obtained from the Oral Microbiome Bank of China. *S. mutans* and its derivatives were cultured in Brain Heart Infusion Broth (Difco, Sparks, MD, USA) at 37 °C within a CO_2_ incubator (5% CO_2_, 95% air) to promote bacterial growth. Sucrose (1% w/v) was introduced to BHI broth to create BHIS broth for biofilm formation. Additional metal ions (ZnSO_4_, CuSO_4_, MnSO_4_, CoSO_4_, MgSO_4_, and NiSO_4_) were added as indicated. Competent *E. coli* DH5α cells [Tsingke Corporation (Beijing, China)] were cultured in LB medium (Difco, Sparks, MD, USA). **Supplementary Table S1** lists the bacterial strains, plasmids, and primers utilized in this study.

### 2.2 Markerless in-frame deletion mutant and complemented strain construction

An in-frame deletion of *zccR* in *S. mutans* was constructed using a cloning-independent mutagenesis approach (Xie et al., 2011). The upstream and downstream regions of the *zccR* gene were first amplified utilizing primers zccRupF/upR and zccRdnF/dnR, respectively, and subsequently ligated to the IFDC2 cassette using primer zccRupF/zccRdnR. The resulting construct was introduced into *S. mutans* UA159 and selected using erythromycin (12 μg/mL on a BHI plate). Primers RupF and zccRdnF amplified the upstream and downstream fragments of *zccR*, which were joined through polymerase chain reaction (PCR) with zccRupF/zccRdnR. These resulting constructs were introduced into erythromycin-selected strains and further selected on BHI plates containing 4 mg/mL *p*-Cl-Phe.

The *zccR* gene and its promoter were amplified and cloned into the shuttle vector pDL278 using the In-Fusion HD cloning kit (TaKaRa Bio, Japan) to generate the recombinant plasmid pDL278*-zccR*. **Supplementary Table S1** contains all the primers used in this study. All constructed strains were verified through PCR analysis and sequencing.

### 2.3 Measurement of cell growth curves

For cell growth assessment, a 96-well plate was inoculated with 10^6^ CFU/mL of mid-log phase bacterial culture, supplemented with various metal cations (ZnSO_4_, MgSO_4_, MnSO_4_, CoSO_4_, CuSO_4_, and NiSO_4_) as needed, in a total culture volume of 200 µL. The growth curve was monitored for 24 h using a microplate reader (BioTek, USA).

### 2.4 Crystal violet test and water-insoluble exopolysaccharide (EPS) determination

A bacterial culture at 10^6^ CFU/mL in mid-log phase was incubated in BHIS broth for 24 h in 48-well or 96-well plates in a CO_2_ incubator (5% CO_2_, 95% air).

For the crystal violet assay, biofilm was washed with PBS, fixed in methanol (15 min), air-dried, and stained with 0.05% crystal violet for 15 min. The stained biofilms were examined under a stereomicroscope (Nikon SMZ800, Nikon Corporation, Japan). Subsequently, 33% acetic acid was applied to dissolve the dye, and the absorbance at 570 nm was recorded (SpectraMax M5, Molecular Devices, USA).

Water-insoluble EPS in biofilms were quantified using the anthrone method, as previously described (Hu et al., 2022). Biofilms were collected from each well, washed twice with phosphate-buffered saline (PBS), and resuspended in 1 mL of 1 M NaOH. The suspension was incubated at 37 °C for 2 h to extract water-insoluble glucans. After centrifugation at 10,000 × *g* for 10 min, an aliquot (200 µL) of the alkali-soluble supernatant was mixed with anthrone-sulfuric acid (600 µL) reagent and heated at 95 °C for 5 min to develop the color. Absorbance of the reaction mixture was measured at 625 nm using a microplate reader (SpectraMax M5, Molecular Devices, USA).

To normalize biofilm biomass to bacterial growth, parallel wells were prepared on the same 24-well plate under identical conditions for EPS quantification and CFU enumeration, respectively. The EPS content was quantified against a glucose standard curve (0–100 μg/mL). For CFU determination, 24 h biofilms were washed twice with PBS, and the adherent cells were scraped, resuspended in PBS by vigorous pipetting and vortexing, serially diluted, and plated onto BHI agar plates. After incubation at 37 °C for 48 h, colonies were counted. The EPS content of each well was normalized to the corresponding viable cell count and expressed as μg EPS per 10^7^ CFU.

### 2.5 Scanning electron microscope (SEM) imaging

*S. mutans* biofilms were established on sterile glass coverslips placed in 24-well tissue culture plates for SEM. After a 24 h incubation, the coverslips were gently washed twice with PBS to remove loosely attached planktonic cells. The biofilms were fixed with 2.5% glutaraldehyde at 4 °C for 4 h and subsequently washed twice with PBS. Samples were dehydrated in a graded ethanol series (30%, 40%, 50%, 60%, 70%, 80%, 90%, and 100%), with each concentration applied for 15 min, dried by critical-point drying with liquid CO_2_ (Leica EM CPD300, Leica Microsystems, Germany), and sputter-coated with approximately 5 nm platinum layer (Leica EM ACE600, Leica Microsystems, Germany; 35mA for 120s) to improve conductivity. Images were acquired using a Quanta 200 scanning electron microscope (FEI, Hillsboro, OR, USA) at an accelerating voltage of 5 kV, working distance of 12 mm, beam current of 0.46 nA, and magnifications of 1,000× and 3,000× to assess biofilm structure.

### 2.6 Deferred antagonism assay

The deferred-antagonism assay was performed as previously described (Tang et al., 2020). To assess *S. gordonii’*s inhibitory effect on *S. mutans* growth, 5 µL of *S. gordonii* overnight culture was spotted onto half-strength BHI agar plates, with or without 150 µM ZnSO_4_. The plates were incubated at 37 °C with 5% CO_2_ for 24 h, after which 5 µL of an overnight *S. mutans* culture was inoculated next to the *S. gordonii* spot. A control group was established by applying 5 µL of catalase solution (1 mg/mL) to the *S. gordonii* colony before introducing *S. mutans* to verify that hydrogen peroxide mediated the inhibition. The *S. mutans* growth inhibition zone was measured after a further 24 h of incubation.

To evaluate *S. mutans*’ inhibitory effect on *S. gordonii, S. salivarius*, and *E. faecalis*, 5 µL of *S. mutans* overnight culture was spotted onto half-strength BHI agar plates, with or without 150 µM ZnSO_4_, and incubated at 37 °C in 5% CO_2_ for 24 h. Subsequently, 500 µL of an overnight indicator strain culture, diluted in 0.75% (w/v) soft agar containing the corresponding concentration of ZnSO_4_, was overlaid onto the plates. Inhibition-zone diameter was measured after an additional 24 h.

### 2.7 Fluorescence *in situ* hybridization (FISH)

FISH was performed to assess the biofilm composition in dual-species biofilms (Hu et al., 2022). *S. mutans* and *S. gordonii* were inoculated into BHIS medium at a final density of 10^6^ cells/mL each and co-incubated for 24 h to form dual-species biofilms. Samples were gently washed twice with PBS and fixed with 4% paraformaldehyde at 4 °C for 12 h. Post-fixation, samples were rinsed twice with distilled water and dried at 46 °C for 10 min. The biofilms were subsequently treated with a lysis buffer containing 100 mM Tris-HCl, 50 mM EDTA, and 30 mg/mL lysozyme, followed by incubation at 37 °C for 20 min. After lysis, samples underwent dehydration using a graded ethanol series (50%, 80%, and 96%) for 3 min each, followed by a 10-min incubation at 46 °C. Hybridization involved applying 20 μL of a fluorescently labeled oligonucleotide probe to each sample, followed by a 90-min incubation at 46 °C in the dark. **Supplementary Table S1** presents species-specific probe sequences. The slides were mounted and examined utilizing a Nikon A1 fluorescence microscope equipped with a 20× lens (Nikon Corporation, Japan). Biofilm quantification was conducted based on coverage area using Image-Pro Plus software (version 6.0, Media Cybernetics, Inc., Silver Spring, USA).

### 2.8 RNA-sequencing (RNA-seq)

For transcriptomic analysis, *S. mutans* was cultured in BHI broth to the mid-exponential phase (optical density at 600 nm [OD_600 nm_] ≈ 0.5), with or without 150 μM Zn^2+^. Following a previously described protocol (Lin et al., 2022), cells were harvested by centrifugation, immediately frozen in liquid nitrogen for 15 min, and stored at –80 °C until further processing. Three biological replicates per condition were sent to Majorbio Co., Ltd. (Shanghai, China) for whole-genome RNA sequencing. Total RNA was extracted and purified using TRIzol^®^ Reagent (Invitrogen), and RNA integrity and concentration were assessed using a 2100 Bioanalyzer (Agilent, USA). Libraries were prepared from 2 μg of total RNA per sample using the TruSeq RNA Sample Preparation Kit (Illumina, USA), followed by double-stranded cDNA synthesis using the SuperScript Double-Stranded cDNA Synthesis Kit (Invitrogen, USA). Libraries were quantified using TBS380 and sequenced with paired-end reads (2 × 150 bp) on an Illumina HiSeq X-Ten platform. Differential expression analysis was performed using the Majorbio Cloud Platform (www.majorbio.com). *P*-values were adjusted for multiple testing using the Benjamini–Hochberg method. Genes with an absolute fold change (absolute) ≥ 2 and adjusted *P*-value < 0.05 were considered differentially expressed. Gene ontology (GO) and Kyoto encyclopedia of genes and genomes (KEGG) enrichment analyses of differentially expressed genes were performed using the R package clusterProfiler (version 4.12.0). The NCBI Sequence Read Archive (SRA) houses the raw sequencing reads under accession number PRJNA1493331.

### 2.9 Quantitative reverse transcription polymerase chain reaction (qRT-PCR)

Transcript levels of selected genes were determined by quantitative reverse-transcription PCR (qRT-PCR). Cells were cultivated to mid-logarithmic phase (OD_600 nm_≈0.5), harvested by centrifugation (4000 g, 4 °C, 10 min), and stored at −80 °C until use. Gene-specific primers (**Supplementary Table S1**) were designed using the Primer3 web tool (https://primer3.ut.ee/). Total RNA was extracted using TRIzol reagent (Invitrogen, USA) as previously described (Xu et al., 2026), and cDNA was synthesized using the HiScript II Q RT SuperMix for qPCR kit (Vazyme, Nanjing, China) following the manufacturer’s instructions. Quantitative PCR was performed on a Step One Plus real-time PCR system (Applied Biosystems, Waltham, MA, USA) with TB Green Premix Ex Taq II (RR820A; Takara Bio, Japan). Transcript levels were normalized to the 16S rRNA gene, and relative expression was calculated using the 2^–ΔΔCt^ method.

### 2.10 Analysis of β-galactosidase activity

Recombinant plasmids carrying promoter*-lacZ* transcriptional fusions were constructed using the shuttle vector pDL278 for β-galactosidase reporter assays in *S. mutans*. The pDL278 backbone was engineered to include the target promoters and *lacZ* operon using an In-Fusion HD cloning kit (TaKaRa Bio, Japan), and all constructs were verified through PCR and sequencing. The reporter plasmids were introduced into the Δ*zccR* mutant to generate four strains: Δ*zccR-zccEp-lacZ*, Δ*zccR-zccRp-lacZ*, Δ*zccR-ldhp-lacZ, and* Δ*zccR-lacZ*. Similarly, they were delivered into the wild-type UA159 to generate UA159*-zccEp-lacZ*, UA159*-zccRp-lacZ*, UA159*-ldhp-lacZ*, and UA159*-lacZ* (**Supplementary Table S1**).

The β-galactosidase activity was measured using the Miller assay with the following procedure. Bacterial cells were harvested from 1 mL of culture (OD_600 nm_ ≈ 0.5) by centrifugation (4,000 rpm, 10 min). The pellet was washed twice with ice-cold PBS and resuspended in 600 μL of Z-buffer. A 300-μL sample was used to measure optical density at 600 nm (A_600_). The remaining 300 μL was mixed with 100 μL chloroform and 50 μL 0.1% SDS, vortexed for 1 min, and incubated at 28 °C for 5 min. Subsequently, 200 μL ONPG (4 mg/mL) was added, mixed, and timed. Upon yellow color formation, 500 μL of 1 M Na_2_CO_3_ was introduced to terminate the reaction. The reaction duration was recorded as T (min). Samples were centrifuged at 12,000 rpm for 5 min. Subsequently, 300 μL of the upper aqueous phase was transferred to a 96-well plate for absorbance measurement at 420 nm (A_420_) and 550 nm (A_550_). The enzyme activity was quantified in Miller units using the following equation.


$$\text{Miller Unit}=1,000\;\times \;\left({\text{A}}_{420}–1.75\;\times {\text{ A}}_{550}\right)/\left({\text{A}}_{600}\times \;0.3\;\times \text{ T}\right)$$

### 2.11 Statistical analysis

All assays were performed in triplicate and independently repeated. Data were analyzed using GraphPad Prism software (version 10; GraphPad, USA). Two-sample t-tests with Holm correction for multiple comparisons were used to compare the means of two groups. Multi-group comparisons were performed using one-way analysis of variance (ANOVA) followed by Holm-corrected pairwise t-tests. A *P* < 0.05 was considered statistically significant.

## 3 Results

### 3.1 ZccR supports *S. mutans* growth under zinc-enriched conditions

To assess the physiological role of the transcriptional regulator ZccR in the zinc efflux system of *S. mutans*, we analyzed the growth kinetics of the wild-type UA159, the isogenic Δ*zccR* mutant, and the complemented strain (*ΔzccR/pDL278-zccR*) under different zinc concentrations (**Figure 1A**).

**Figure 1:**
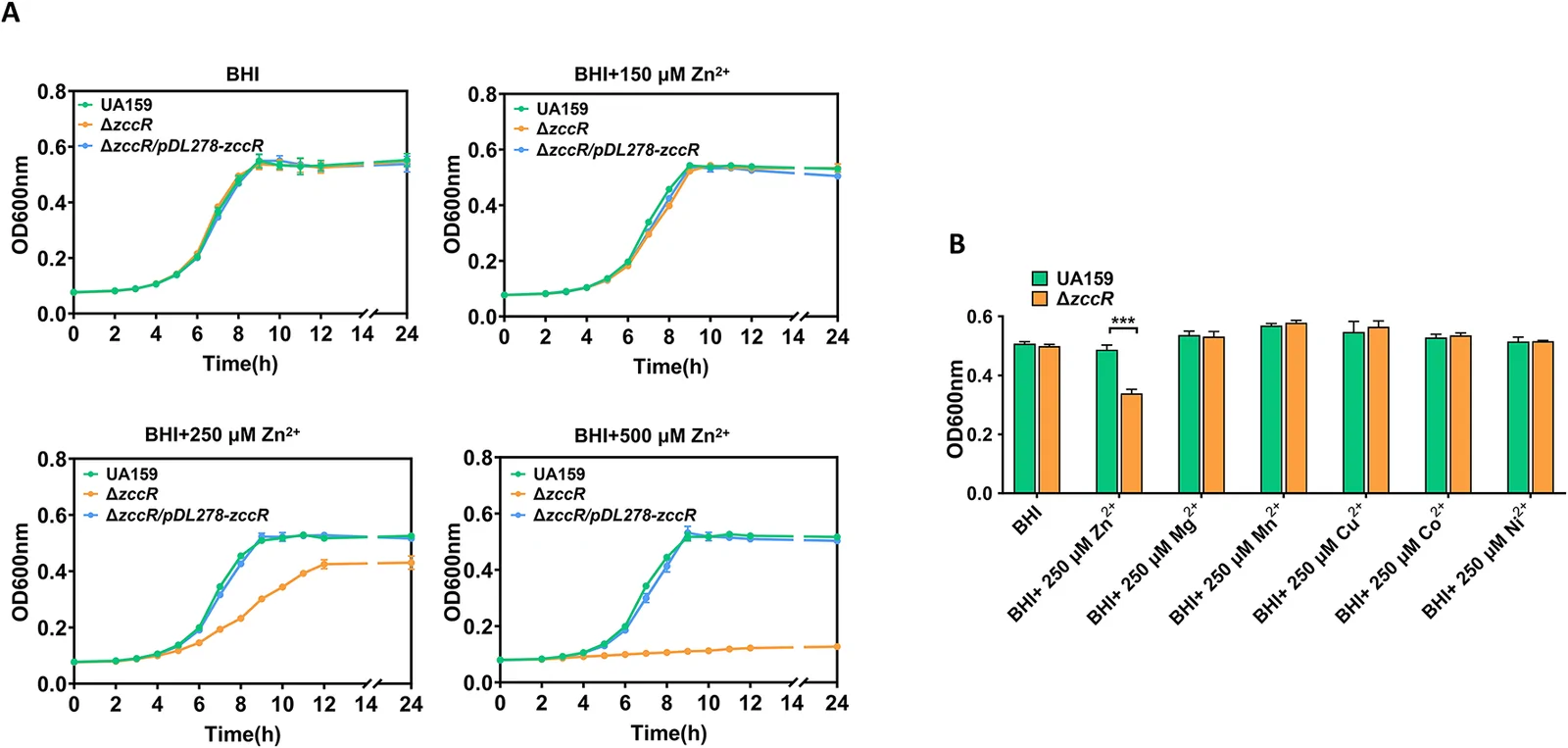
Effects of *zccR* deletion on the bacterial growth of *S. mutans.* **(A)** Growth curves of *S. mutans* strains grown in the BHI medium supplemented with different concentrations of Zn^2+^ (0, 150, 250, and 500 μM). Curves indicate the average and SD of at least three independent biological replicates. **(B)** Optical density at 600 nm (OD_600 nm_) of *S. mutans* strains cultivated in the BHI medium supplemented with different ions for 24 h. The results indicated the mean ± standard deviation of three replicates. Two-sample t-tests with Holm correction were utilized to compare the differences in absorbance between ΔzccR mutant and wild-type UA159 strains. (***P < 0.001).

In unsupplemented BHI medium, deletion of *zccR* did not cause an apparent growth defect. However, as the zinc concentration in the medium increased, the mutant exhibited progressively more severe growth inhibition. The Δ*zccR* mutant exhibited a slight growth delay compared to the wild-type and complemented strains at 150 μM Zn^2+^. At 250 μM, the mutant exhibited significantly impaired growth, characterized by a prolonged lag phase and a lower stationary-phase optical density (**Figure 1B**). At 500 μM, the mutant barely grew. These findings indicate that ZccR is crucial for *S. mutans*’ optimal growth in zinc-rich environments, and that zinc’s inhibitory impact on bacterial growth is dose-dependent.

### 3.2 Absence of *zccR* reduced *S. mutans* biofilm formation under zinc-enriched conditions

As a major cariogenic pathogen, biofilm formation is a critical virulence determinant of *S. mutans*. We assessed biofilm formation by quantifying total biomass using crystal violet staining and water-insoluble EPS production using the sulfuric acid-anthrone colorimetric method. Under zinc-enriched media, the mutant exhibited a marked reduction in biofilm biomass and EPS production, with almost no biofilm formation at 500 μM Zn^2+^ (**Figures 2A–C**). Notably, at 150 μM Zn^2+^, planktonic growth of the mutant was only marginally affected compared with the wild type (**Figure 1A**), whereas both total biofilm biomass and EPS production were significantly reduced under biofilm conditions. We assessed whether the impairment was linked to changes in glucan synthesis gene expression by analyzing the transcript levels of *gtfB* and *gtfC*, which are critical for extracellular polysaccharide synthesis. No significant differences were observed between the mutant and wild type under the tested zinc concentration (**Supplementary Figure S1**).

**Figure 2:**
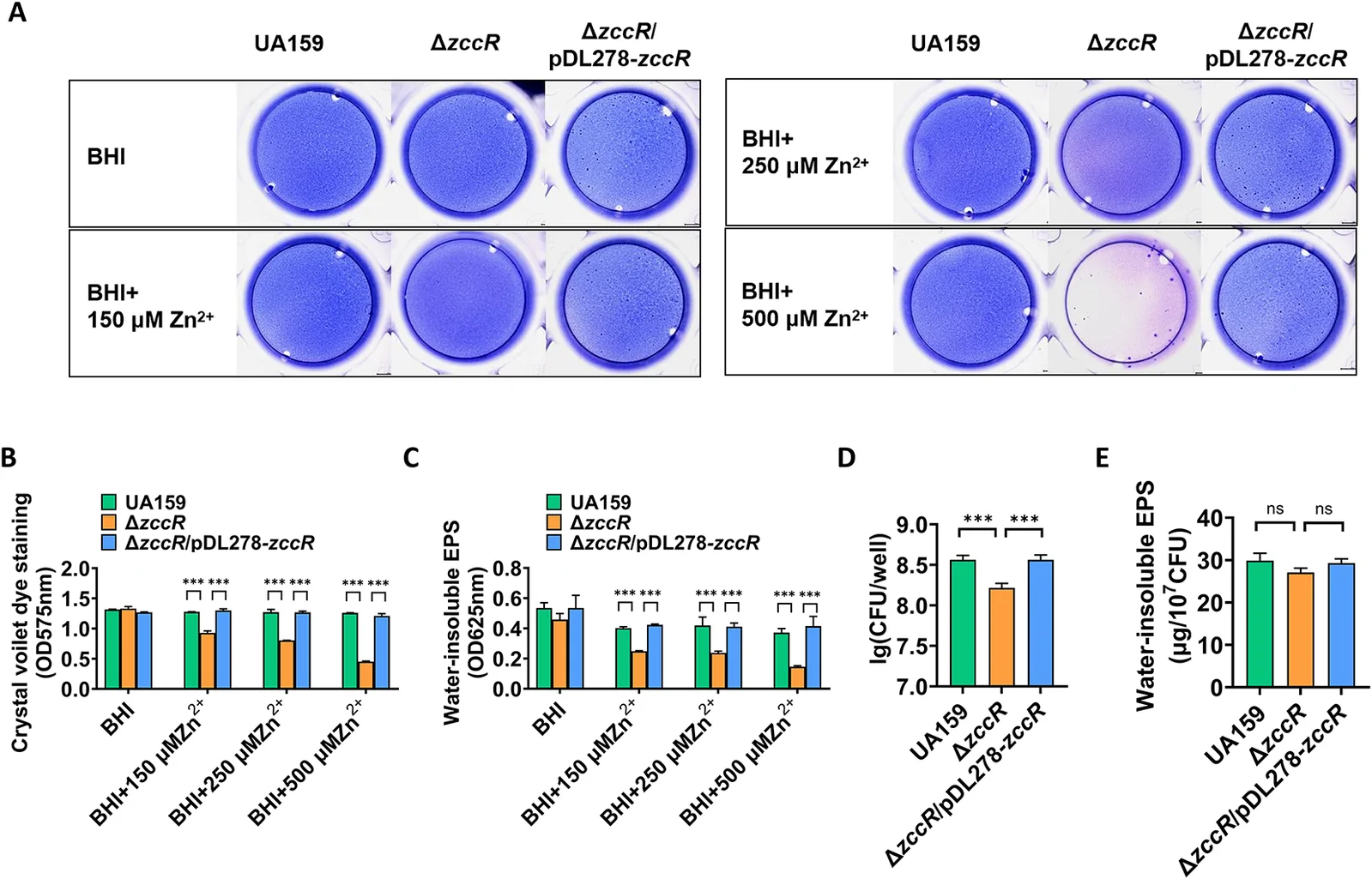
Impact of *zccR* gene deletion on *S. mutans* biofilm development. **(A)** Crystal violet staining of biofilms. **(B)** Quantification of biofilm was performed with crystal violet staining. **(C)** The water-insoluble EPS content in the biofilms was measured by the anthrone method. **(D)** CFU counts in the 24 h biofilms of the wild-type UA159, Δ*zccR* mutant, and complemented strains under 150 μM Zn^2+^ treatment. **(E)** The water-insoluble EPS were normalized to CFU counts of the 24 h biofilms formed by the wild-type UA159, Δ*zccR* mutant, and complemented strains under 150 μM Zn^2+^ treatment. Each sample was measured in triplicate. The results are presented as the mean ± standard deviation. Group differences were evaluated using one-way ANOVA followed by Holm-corrected pairwise t-tests. (***P < 0.001; ns, not significant).

Because reductions in biofilm biomass and EPS content could reflect impaired growth, we further normalized water-insoluble EPS production to viable cell counts (CFU) at 150 μM Zn^2+^. The EPS content was quantified from a glucose standard curve (y = 415.38x − 24.964, R^2^ = 0.9921), with parallel wells used for CFU enumeration. The CFU count of the Δ*zccR* mutant was significantly lower than that of the wild-type and complemented strains (**Figure 2D**), whereas the EPS normalized to CFU (μg/10^7^ CFU) exhibited only a downward trend with no significant difference (**Figure 2E**). These results indicate that the apparent EPS reduction in the mutant at 150 μM Zn^2+^ predominantly reflects the smaller viable biofilm population rather than a specific defect in EPS biosynthesis per cell.

To further visualize the structural changes in biofilms, SEM was performed. **Figure 3** illustrates that higher zinc concentrations significantly reduced biofilm formation in the Δ*zccR* mutant, with decreased EPS and a disorganized structure. Conversely, the wild-type and complemented strains retained dense biofilms with cells embedded within the EPS. The biofilm morphology of the wild-type and complemented strains exhibited minimal variation across different zinc concentrations.

**Figure 3:**
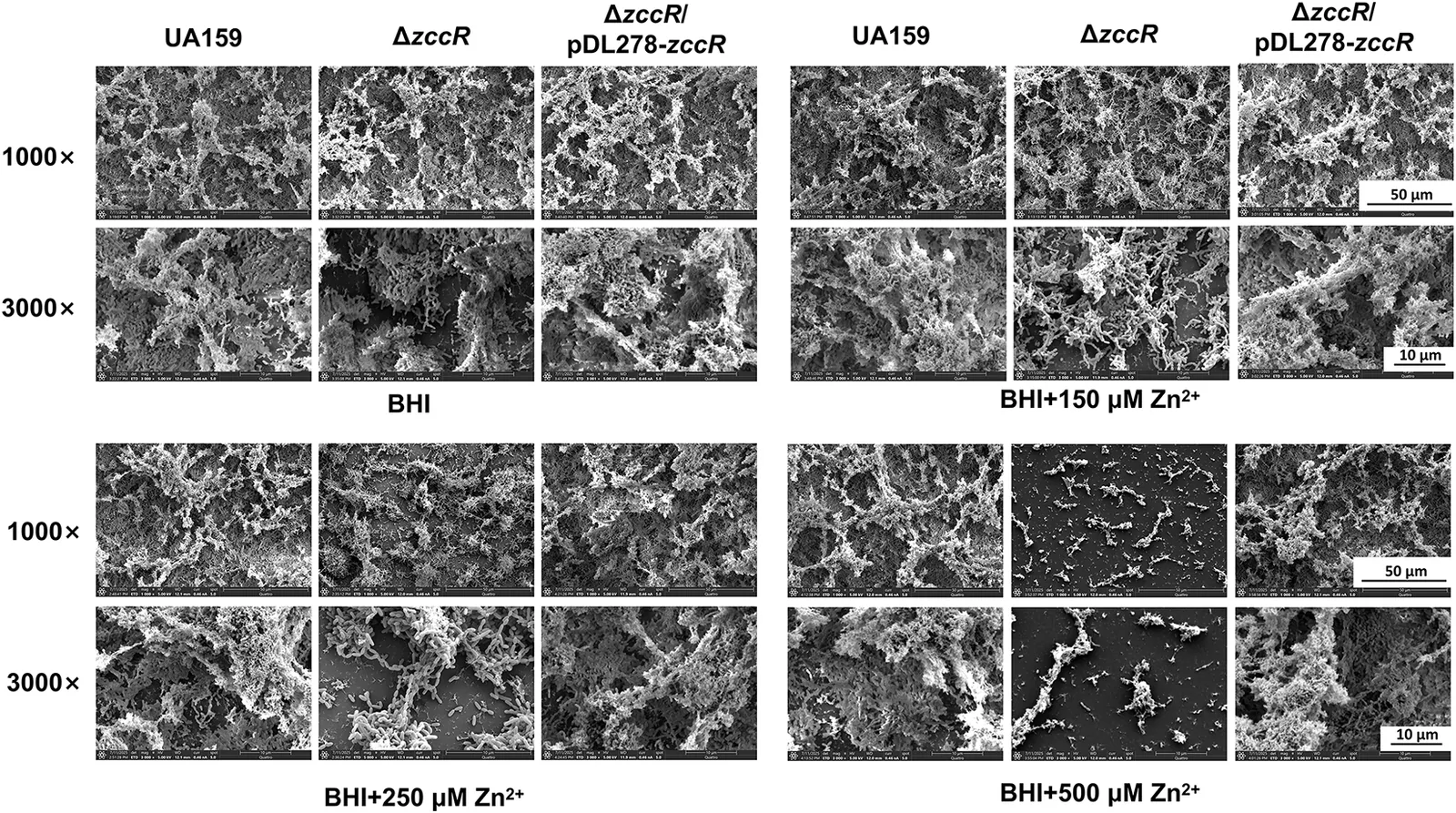
SEM analysis of *S. mutans* biofilms formed under different Zn^2+^ concentration culture conditions for 24 h. The specimens were examined at an accelerating voltage of 5 kV, and images were captured at magnifications of 1,000× and 3,000×.

### 3.3 Deletion of *zccR* modifies the composition of dual-species biofilms of *S. mutans* and *S. gordonii* in zinc-enriched environments

The ecological plaque hypothesis proposes that dental caries result from a detrimental shift in the balance of oral microbiota in response to environmental changes within the dental niche. We therefore analyzed the phenotype of dual-species biofilms formed by the Δ*zccR* mutant and the commensal *S. gordonii* to assess the effect of ZccR on ecological balance. FISH analysis revealed that, in media supplemented with 150 μM Zn^2+^, *S. gordonii* gained a significant competitive advantage over the Δ*zccR* mutant compared with the other groups. In unsupplemented medium, however, deletion of *zccR* had no marked effect on the biofilm composition (**Figure 4A**). Semi-quantitative analysis further revealed that, under zinc-supplemented conditions, the proportion of wild-type *S. mutans* increased relative to that in unsupplemented medium, indicating greater sensitivity of *S. gordonii* to zinc stress. Notably, the Δ*zccR* mutant reversed this trend, indicating that zinc, in the absence of ZccR, tips the competitive balance in favor of *S. gordonii* (**Figure 4B**).

**Figure 4:**
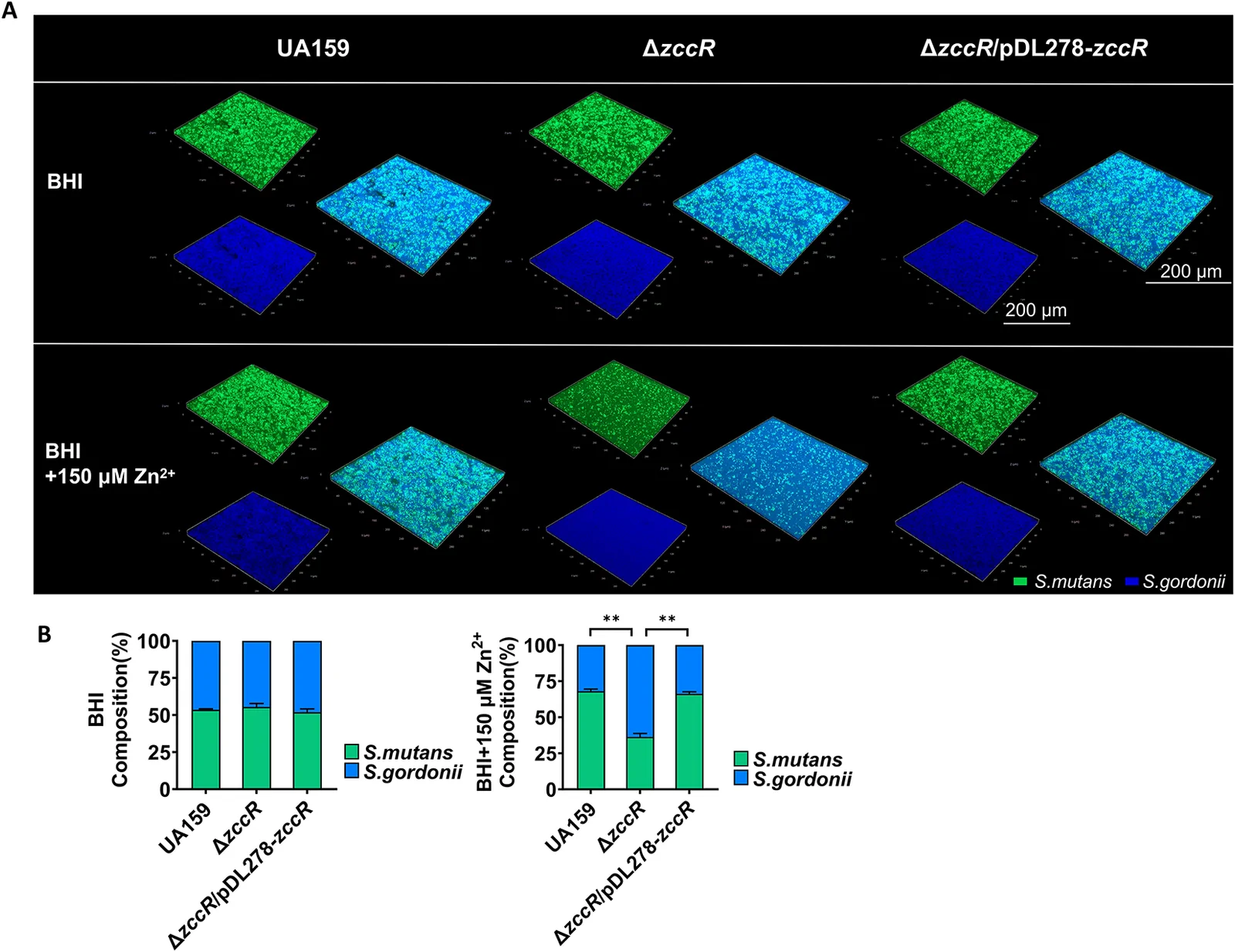
*S. mutans* and *S. gordonii* dual-species biofilms revealed using FISH: **(A)** 24 h dual-species biofilms formed by the wild type, Δ*zccR* mutant, and the complemented strain with *S. gordonii* in BHI medium supplemented with 1% (w/v) sucrose, respectively (*S. mutans* was stained green and *S. gordonii* was stained blue). **(B)** Composition ratio of *S. mutans* and *S. gordonii* in dual-species biofilms according to coverage area. Results are the average of three randomly selected positions in each sample. Data are presented as mean ± standard deviation. Group differences were evaluated by one-way ANOVA followed by Holm-corrected pairwise t-tests. (**P < 0.01).

To further corroborate these findings, a plate competition assay was performed to assess changes in the competitive interaction between *S. gordonii* and *S. mutans* under varying zinc concentrations. When *S. gordonii* served as the pioneer strain, inhibition zones exhibited no significant difference between the wild-type and Δ*zccR* mutant without supplementary zinc. However, upon addition of 150 μM Zn^2+^, the inhibition zone around the Δ*zccR* mutant was significantly larger than that around the wild-type. Catalase eliminated the inhibition zones for both strains, indicating that the differential sensitivity was attributed to differences in oxidative stress tolerance (**Figure 5A**). Conversely, when *S. mutans* was the pioneer strain, the Δ*zccR* mutant exhibited a markedly weakened inhibitory effect on *S. gordonii* under zinc-supplemented conditions. This reduction was not reversed by catalase, suggesting that the impaired inhibition was not attributed to altered oxidative stress resistance in *S. mutans* (**Figure 5B**). To determine whether the attenuated antagonism of the Δ*zccR* mutant was specific to *S. gordonii*, we extended the plate competition assay to *S. salivarius* and the non-streptococcal *E. faecalis*. In both cases, the Δ*zccR* mutant produced narrower inhibition zones than UA159 and the complemented strain under 150 μM Zn^2+^, indicating that the phenotype extends beyond the *S. mutans*–*S. gordonii* pair (**Supplementary Figure S2**).

**Figure 5:**
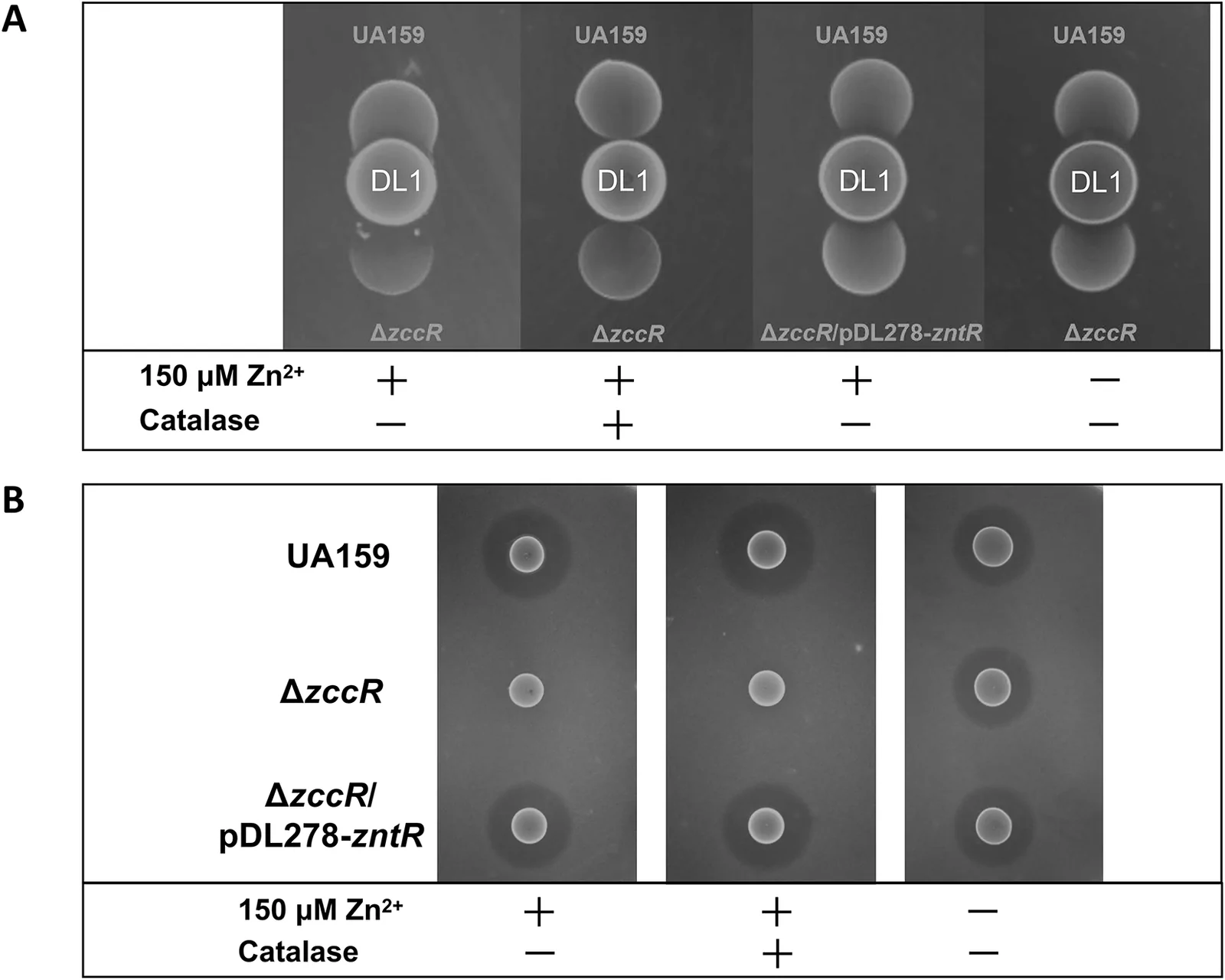
Deferred-antagonism assay of *S. mutans* and *S. gordonii*. **(A)** Plate competition assay of *S. mutans* wild-type, *zccR mutant*, and complemented strains with *S. gordonii* (DL1). *S. gordonii* strains were the initial colonizers, incubated for 24 h before the secondary colonizers. **(B)** *S. mutans* were spotted on BHI agar and incubated overnight. Cultures of *S. gordonii* were added to soft BHI agar and used to overlay the preformed *S. mutans* spots. Zones of inhibition were measured 24 h after the addition of the assay.

Collectively, these findings indicate that under zinc-rich environments, the deletion of *zccR* reduces *S. mutans*’ oxidative tolerance and its capacity to inhibit *S. gordonii* and other oral bacteria, including the commensal *S. salivarius* and the non-streptococcal Gram-positive species *E. faecalis*, and alters *S. mutans-S. gordonii* dual-species biofilm composition to favor the commensal partner.

### 3.4 Deletion of *zccR* globally affects the *S. mutans* transcriptome in zinc-rich environments

To further investigate the association between *zccR* gene and the observed mutant phenotype, we performed genome-wide transcriptional analysis using RNA-seq. In medium supplemented with 150 μM Zn^2+^, the Δ*zccR* mutant exhibited differential expression of 89 genes compared to the wild-type, including 67 upregulated and 22 downregulated genes.

The zinc-efflux P-type ATPase gene *zccE/smu.2057c* was significantly downregulated (0.01-fold), whereas ion-uptake transporter cluster (*smu.995-998*, 3.0-3.8-fold) and copper export-related genes (*copY/smu.424*, 4.32-fold; *copA/smu.426*, 2.98-fold; *copZ/smu.427*, 2.47-fold) were upregulated. Nitrogen metabolism-related genes, including glutamine synthetase (*glnA/smu.364*, 3.8-fold), PII-type signal transduction protein (*glnB/smu.1657c*, 2.2-fold), and the ammonium transporter (*nrgA/smu.1658*, 4.5-fold), were significantly upregulated, as were genes involved in cystine transport (*tcyDEFGH/smu.933-936*, 6–8-fold) and their LysR-type transcriptional regulator HomR (*smu.930c*, 2.2-fold). Among carbohydrate metabolism-related genes, the lactose metabolism gene cluster (*lacA-G/smu.1490-1496*, 3-15-fold; *lacR/smu.1498*, 3.14-fold) was upregulated, while the sucrose-specific PTS transporter (*scrA/smu.1841*, 0.41-fold) and the maltose/maltodextrin ABC transporter (*msmX/smu.1571*, 0.493-fold) were downregulated, indicating preferential galactose utilization. Additionally, bacteriocin family genes (*nlmABD*, 2–3-fold) and CRISPR-associated protein genes (*cas2/smu.1753c, cas7c/smu.1758c, cas4/smu.1760c, cas8c/smu.1761c, cas5c/smu.1763c, cas3/smu.1764c*;2.7–3.7-fold), were upregulated, whereas mutanobactin A system protein genes (*mubA/smu.1342* and *mubX/smu.1366*), two FAD-dependent oxidoreductase (*smu.1410; smu.1117*), and several multidrug transport system genes (*smu.524/mdlA, smu.525/mdlB, and smu.71*) were downregulated (**Figure 6A**; **Supplementary Tables S2**–**S4**). To validate the transcriptomic findings, RT-qPCR was performed on representative differentially expressed genes under the same zinc-enriched condition (150 μM Zn^2+^). The direction of the RT-qPCR changes was consistent with RNA-seq data (**Supplementary Figure S3**), supporting the transcriptomic conclusions.

**Figure 6:**
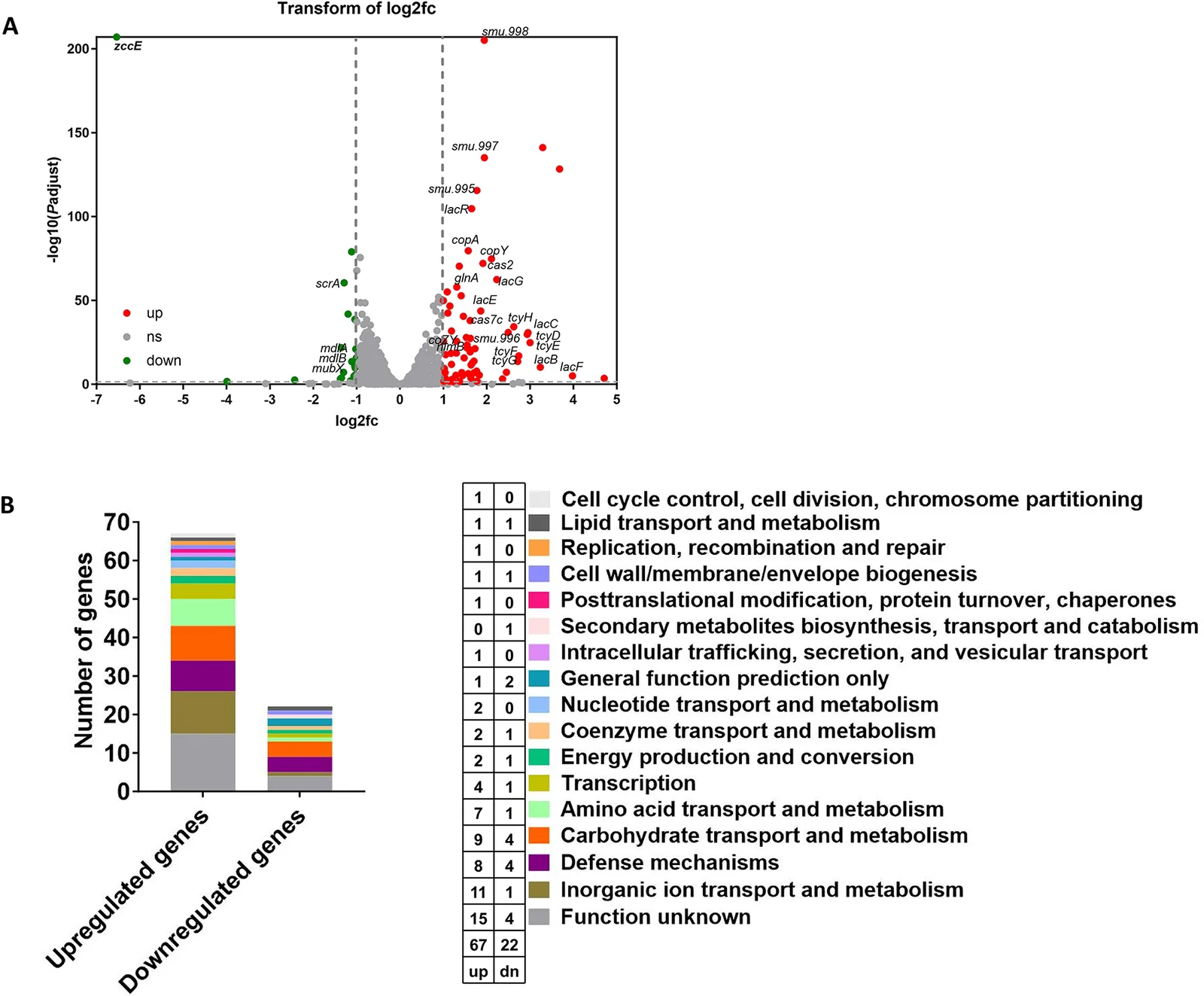
Global effects of the *zccR* gene mutation for *S. mutans* on transcript levels. **(A)** Volcano plot of differentially expressed genes (DEGs) between wild-type and Δ*zccR* strain cultured with 150 μM Zn^2+^ from the transcriptomes. **(B)** COG database function annotation and classification of the DEGs.

Analysis of COG functional classification revealed that upregulated genes were predominantly enriched in inorganic ion transport and metabolism, defense mechanisms (primarily the CRISPR-Cas system), carbohydrate transport and metabolism (specifically lactose metabolism), and amino acid transport and metabolism. Conversely, the downregulated genes were primarily associated with defense mechanisms, such as multidrug transport systems and carbohydrate transport and metabolism (e.g., scrA) (**Figure 6B**).

GO enrichment analysis identified significantly enriched biological processes, including D-allose, galactose, and lactose catabolism via tagatose-6-phosphate, siderophore-iron import, and defense response to virus, and the molecular function of galactose-6-phosphate isomerase activity (**Figure 7A**). KEGG pathway enrichment revealed significant representation in sulfur, galactose, glyoxylate, and dicarboxylate metabolism, and ABC transporters (**Figure 7B**).

**Figure 7:**
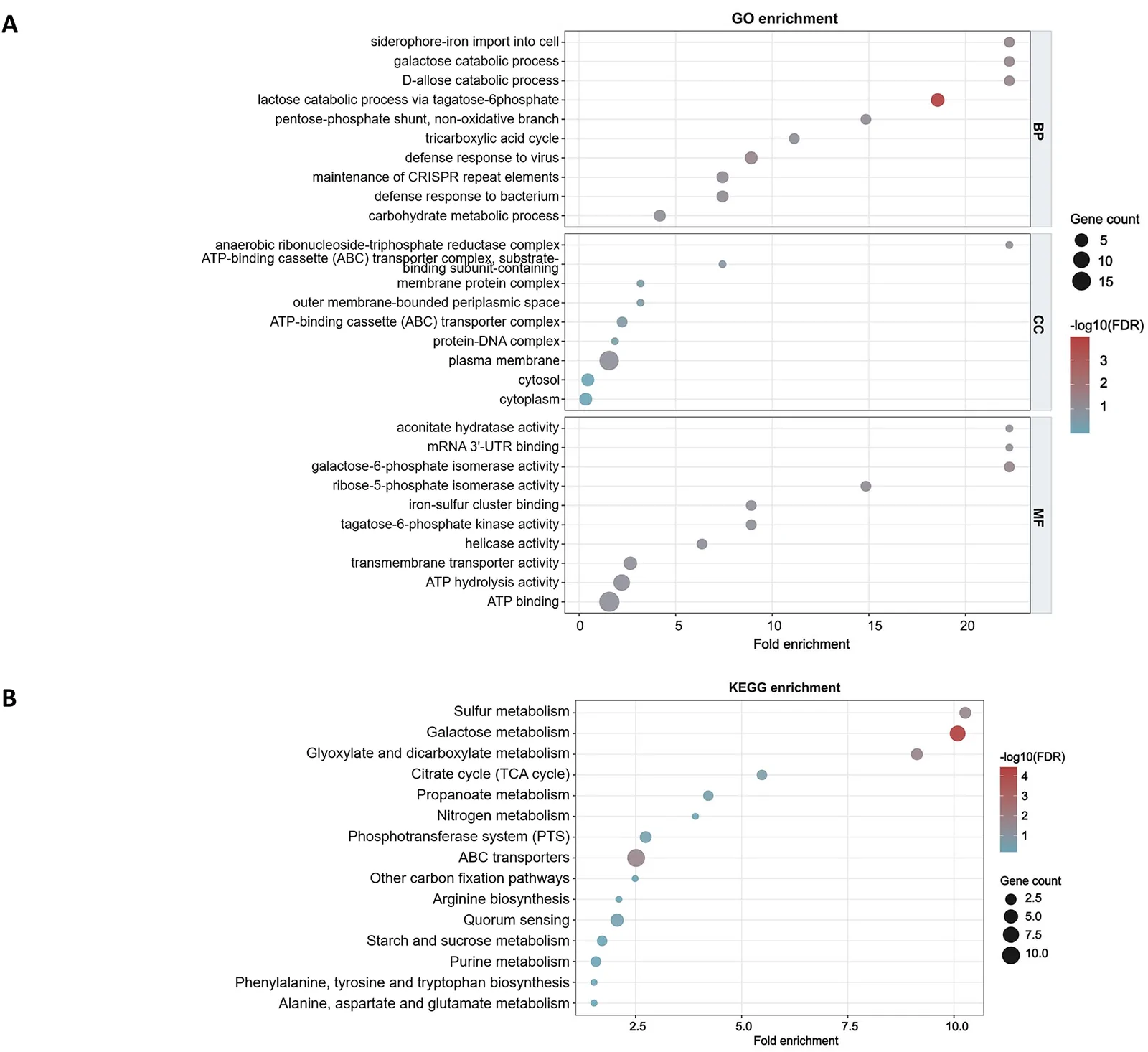
Functional enrichment analysis of the identified differentially expressed genes (DEGs) using GO and KEGG pathways. **(A)** The bubble graph of GO functional enrichment analysis. **(B)** The bubble graph of KEGG functional enrichment analysis. GO, Gene Ontology; KEGG, Kyoto Encyclopedia of Genes and Genomes. Enrichment analysis was performed using the R package clusterProfiler (version 4.12.0).

These findings indicate that deleting *zccR* significantly alters transcription of genes associated with key physiological processes, including carbon and nitrogen metabolism, metal ion transport, sulfur metabolism, and defense systems, under zinc-enriched conditions. Because ZccR is a transcriptional regulator, we sought to distinguish direct regulatory effects of ZccR from the indirect consequences of intracellular zinc accumulation resulting from impaired zinc efflux. We therefore compared the transcriptional changes of the Δ*zccR* mutant grown in zinc-enriched medium versus unsupplemented medium. Notably, nearly all differentially expressed genes identified in the mutant versus wild-type comparison under zinc-enriched conditions exhibited the same directional trends in this additional comparison, with the sole exception of *smu.1284c*, *smu.1942c*, *smu.1956c*, and *zccE*, although the significance levels sometimes differed (**Supplementary Table S2**). Our observations indicate that the transcriptional changes in the mutant are predominantly due to intracellular zinc accumulation, rather than direct regulation by ZccR, aside from the effects of *zccE* itself.

### 3.5 ZccR bidirectionally regulates zccE and its own promoter transcription

*In vitro* studies have revealed that the MerR-type transcription factor ZccR positively regulates *zccE* (Ganguly et al., 2022). However, its autoregulatory pattern remains unclear. We therefore constructed *lacZ* reporter strains to investigate the regulatory effect of ZccR on its own promoter and validate its control over *zccE* transcription *in vivo*. The results revealed that in the Δ*zccR* mutant, *zccE* promoter activity was repressed, whereas *zccR* promoter activity was enhanced (**Figure 8A**). Based on these observations, we propose that ZccR activates *zccE* promoter expression, aligning with Ganguly et al (Ganguly et al., 2022), and represses its own promoter.

**Figure 8:**
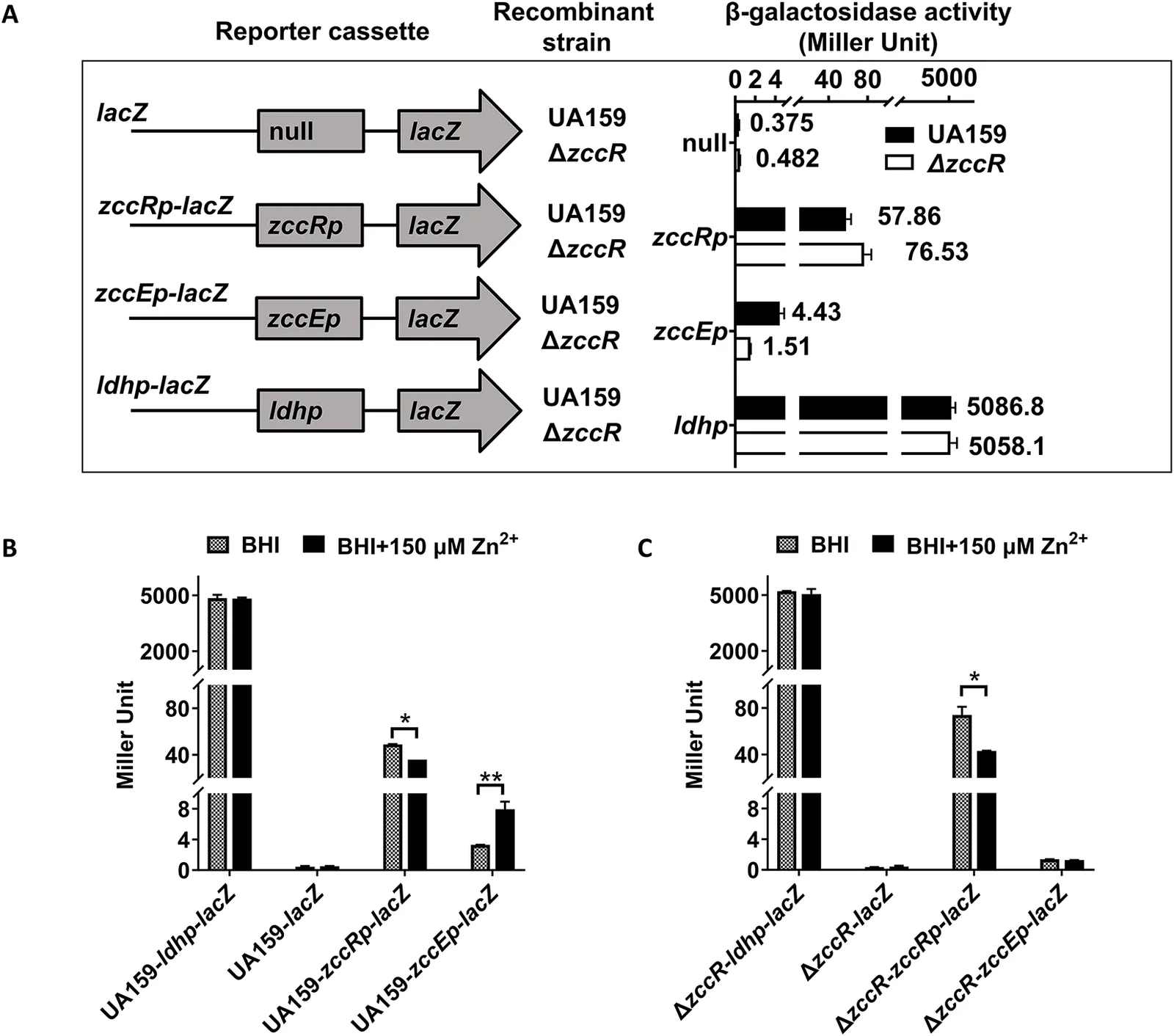
Promoter activities of *zccE* and *zccR* were detected by a series of LacZ reporter strains. **(A)** Left column: each co-expression plasmid used to generate the following two-group reporter strains: Δ*zccR-zccRp-lacZ*, Δ*zccR-zccEp-lacZ*, Δ*zccR-ldhp-lacZ*, Δ*zccR-lacZ*, UA159*-zccRp-lacZ*, UA159*-zccEp-lacZ*, UA159*-ldhp-lacZ*, and UA159*-lacZ*. Null promoter*-lacZ* and *ldhp-lacZ* were used as negative and positive controls, respectively. Right column: The activities of β-galactosidase from three independent assays were measured and presented as Miller units. **(B)** β-Galactosidase activity was measured in *S. mutans* wild-type reporter strains in the presence or absence of 150 μM Zn^2+^. **(C)** β-Galactosidase activity was measured in *zccR* mutant reporter strains in the presence or absence of 150 μM Zn^2+^. The means of three independent replications were indicated with standard deviations. Two-sample t-tests with Holm correction were used to compare the differences. (*P < 0.05; **P < 0.01).

We further examined the modulatory role of Zn^2+^ on these promoter activities. In the wild-type strain, zinc supplementation stimulated *zccE* promoter activity; whereas this stimulatory effect was abolished in the Δ*zccR* mutant, indicating that zinc-mediated activation of *zccE* transcription is ZccR-dependent. Whereas zinc inhibited *zccR* promoter activity in the wild-type and *zccR-*deficient strains, suggesting that zinc’s suppression of *zccR* transcription is independent of ZccR (**Figures 8B, C**).

## 4 Discussion

This study investigated the role of the MerR-type transcriptional regulator encoding gene *zccR* in *S. mutans* growth, biofilm formation, water-insoluble EPS production, intermicrobial competitiveness, and transcriptomic responses. Additionally, the regulatory role of ZccR in the transcription of *zccE* and its own promoter was examined. We found that *zccR* in *S. mutans* is required for optimal growth, biofilm formation, water-insoluble EPS production, and competition against the primary inhabitant *S. gordonii* in the presence of Zn^2+^. Furthermore, *S. mutans* ZccR triggers widespread transcriptional remodeling under zinc exposure. Additionally, ZccR activates *zccE* promoter expression and represses its own promoter expression.

Metal ions, including zinc ions, are essential for numerous reactions, but excessive metal concentration can be toxic (Chandrangsu et al., 2017). In the presence of Zn^2+^, the *zccR* mutant can lead to a significant increase in intracellular zinc accumulation in *S. mutans* (Ganguly et al., 2022). This may partly explain the reduced biological activity, biofilm formation, and *zccR* mutant EPS production in the presence of Zn^2+^. Similarly, there is a correlation among these three phenotypic changes. Biofilm biomass suppression is partly attributable to impaired growth, and the reduction in water-insoluble EPS production is, subsequently, partly attributable to the reduced biofilm biomass formed under the same zinc exposure. Δ*zccR* mutant cultured with 150 μM Zn^2+^ produced much less water-insoluble EPS, whereas, when normalized to viable cell counts, the normalized EPS yield exhibited only a non-significant downward trend. Notably, although planktonic growth of the mutant was only marginally affected at this concentration, viable cell counts within biofilms were markedly reduced, suggesting that the zinc-mediated defect preferentially compromises biofilm-associated survival or retention. These results indicate that the significant reduction in total EPS content in the mutant largely reflects the smaller viable biofilm population rather than per-cell EPS biosynthesis. Accordingly, RNA-seq analysis revealed that the glucan-synthesis genes *gtfB*, *gtfC*, and *gtfD*, and the adhesin gene *spaP*, exhibited only modest downward trends that did not meet the predefined threshold for differential expression (≥ 2-fold change; adjusted *P* < 0.05). Similarly, the sortase-encoding gene *srtA* and the biofilm-regulatory genes *vicR, comD*, and *luxS* remained largely unchanged or exhibited only marginal increases, none of which met the differential-expression threshold (**Supplementary Table S3**). However, a subtle per-cell reduction in EPS synthesis cannot be fully excluded, as the normalized EPS yield still trended downward. Previous studies have demonstrated that Zn^2+^ can suppress glucosyltransferase (Gtf) activity by covalently modifying amino acid sulfhydryl groups (Zhang et al., 2021), raising the possibility that zinc inhibits Gtf activity at the post-transcriptional level in *S. mutans*. However, direct measurements of Gtf enzyme activity and protein-level effects of zinc on Gtfs are needed to verify this hypothesis.

Dental caries development is closely associated with the homeostasis of dental plaque biofilms, in which microbial interactions are crucial for maintaining community balance (Huang et al., 2018; Lamont et al., 2018). Our findings indicate that loss of the MerR-family zinc-efflux regulator ZccR reduced *S. mutans* competitiveness under zinc-enriched conditions. Two phenotypes stood out experimentally: weaker inhibition of *S. gordonii* and reduced tolerance to H_2_O_2_-mediated oxidative stress; both occurred at zinc levels that barely slowed planktonic growth. The results from the competition assays indicate that when *S. gordonii* served as the pioneer strain, the inhibition zones of the Δ*zccR* mutant expanded, and this effect was abolished by the addition of catalase, indicating a reduced tolerance to oxidative stress (Kreth et al., 2008). Several transcriptomic changes are consistent with this phenotype. The mutanobactin synthetase and transporter genes *mubA* and *mubX* decreased to approximately 0.4–0.5-fold of wild-type levels. Mutanobactin, a hybrid polyketide/non-ribosomal peptide (PK/NRP) secondary metabolite, is synthesized by the *mub* gene cluster located on the genomic island TnSmu2 of *S. mutans* (Wu et al., 2010). Studies demonstrate that mutanobactin serves as a critical molecule for bacterial defense against oxidative damage; *mub* knockout mutants exhibit significantly increased sensitivity to oxygen and hydrogen peroxide, significantly reduced competitiveness when co-cultured with H_2_O_2_-producing symbionts, and impaired biofilm maturation under aerobic conditions (Rainey et al., 2019). Meanwhile, the iron-chelate uptake operon *smu.995–smu.998* increased 3.0–3.8-fold. Excess intracellular Fe may promote the Fenton reaction and generate hydroxyl radicals that overwhelm cellular antioxidant defenses and cause oxidative damage to DNA, proteins, and membrane lipids (Wu et al., 2010; Ganguly et al., 2018). Two FAD-dependent oxidoreductases (*smu.1117* and *smu.1410*) were also downregulated, which may further compromise reductive detoxification, although the direct contribution of these enzymes to H_2_O_2_ tolerance in *S. mutans* remains undefined (Yu et al., 2023). Collectively, these transcript-level associations provide plausible, but not yet demonstrated, mechanistic links between zinc dyshomeostasis, mutanobactin, and iron-handling pathways and the observed oxidative-stress phenotype. Direct measurements of mutanobactin production, intracellular iron and zinc pools, and H_2_O_2_-tolerance assays in defined genetic backgrounds are needed to distinguish among these possibilities.

A notable finding was that *S. mutans* bacteriocin genes (*nlmA*, *nlmB*, and *nlmD*) were upregulated (2.0-3.1-fold), yet the Δ*zccR* mutant inhibited *S. gordonii* less effectively than the wild type. Production of nonlantibiotic mutacin requires coordinated transcription, leader-peptide processing, ABC-dependent export, and extracellular activity (Merritt and Qi, 2012; Watanabe et al., 2021). Expression of the dedicated exporter *nlmTE* was not significantly altered, so a simple transcriptional defect in export is unlikely (Hale et al., 2005). One possibility is that increased *nlm* transcript levels do not translate into stronger antagonism if peptide maturation or secreted activity is impaired by excess intracellular zinc; alternatively, zinc may disrupt membrane physiology or cellular energy status, thereby limiting bacteriocin deployment. These explanations remain speculative, and direct measurements of mature mutacin production and activity under defined zinc conditions are needed. Thus, the competitive defect of Δ*zccR* against *S. gordonii* cannot be inferred from bacteriocin transcript levels alone.

*S. mutans zccR* activates widespread transcriptional remodeling under zinc exposure. Notably, zinc interacts with diverse intracellular metal-binding proteins in bacterial cells (Chandrangsu et al., 2017). Excessive zinc accumulation may disrupt protein functions to induce extensive regulatory and compensatory transcriptional changes in *S. mutans*. The Δ*zccR* mutant exhibited a notable upregulation of the transcriptional regulator HomR and the L-cystine uptake-associated ABC transporter operon *tcyDEFGH*. Cysteine-rich metallothioneins (MTs) chelate zinc and other metals, participating in intracellular metal homeostasis regulation and antioxidant capacity (Subramanian Vignesh and Deepe, 2017), whereas protein glutathionylation regulates antioxidant protection, detoxification, signal transduction, and cell proliferation in *S. mutans* (Li et al., 2020; Yu et al., 2023). *S. mutans* synthesizes glutathione (GSH) through glutamylcysteine synthase (GshAB), with cysteine availability being the rate-limiting factor (Vergauwen et al., 2013). Accordingly, enhanced L-cystine uptake may represent a compensatory mechanism that restores thiol buffering for metal homeostasis and oxidative defense under zinc stress. Consistent with the Irving–Williams series, excess Zn^2+^ can outcompete most divalent metals (except Cu and Ni) for metalloprotein binding, resulting in zinc-driven mismetallation, as reported in other *streptococci* (Irving and Williams, 1953; McDevitt et al., 2011; Chandrangsu et al., 2017; Makthal and Kumaraswami, 2017). Consistently, the upregulation of *glnA*, *glnB*, and *nrgA* may therefore compensate for Zn^2+^-impaired GlnA activity, which depends on Mn^2+^ or Mg^2+^, thereby preserving nitrogen homeostasis under zinc stress (Ardin et al., 2014; Mowbray et al., 2014). Additionally, Zn^2+^-induced metal displacement causes functional iron deficiency, leading to compensatory upregulation of iron-import genes *smu.995–smu.998*. Beyond metal and nutrient homeostasis adjustment, zinc stress also significantly remodels bacterial metabolism and defense pathways in the Δ*zccR* mutant. The repression of *scrA* and *msmX* and robust induction of the *lac* operon (*lacA–lacG* and *lacR*) indicate a preferential shift toward lactose and galactose catabolism through the tagatose-6-phosphate pathway. This metabolic rewiring represents a low-efficiency carbon bypass that sustains cellular energy supply when canonical glycolysis may be perturbed by zinc stress. However, zinc stress significantly altered the expression of CRISPR-associated defense genes, significantly upregulating multiple type I-C CRISPR genes (*cas2, cas3, cas4, cas5c, cas7c*, and *cas8c*). Beyond canonical adaptive immunity, the *S. mutans* CRISPR–Cas system contributes to cellular stress tolerance, including defense against oxidative and membrane stress (Serbanescu et al., 2015). Whether CRISPR–Cas functionality directly regulates zinc stress resistance in *S. mutans* remains to be determined.

This study demonstrates that ZccR activates the effector gene *zccE* and represses its own promoter, a regulatory pattern also observed among *S. mutans* transcription regulators (Chattoraj et al., 2011). A similar dual activator–repressor pattern of MerR-family proteins has been reinforced by works that MerR regulators remain operator-bound in apo and effector-bound states and switch between repression and activation through ligand-driven conformational change and promoter-DNA distortion (Chang et al., 2015; Yang et al., 2021). Zinc stimulated *zccE* transcription only in the presence of ZccR, indicating that Zn^2+^ is the allosteric signal for ZccR-dependent activation. Conversely, zinc repressed the *zccR* promoter even in the Δ*zccR* background. We therefore hypothesize that a second, ZccR-independent zinc-responsive regulatory input acts at this locus. A plausible candidate is AdcR, the MarR-family zinc-uptake regulator of *S. mutans*, which senses elevated zinc and down-regulates zinc-import genes, thereby complementing ZccR-mediated efflux in zinc homeostasis (Pan et al., 2021). Alternatively, zinc-dependent repression observed in the *ΔzccR* background may occur indirectly—for instance, through zinc-induced changes in other metal-responsive or global regulators—rather than by a dedicated zinc-sensing repressor acting directly on this promoter. Such dual input would couple efflux capacity to regulator abundance and allow zinc itself to limit unnecessary *zccR* expression when efflux demand is already high, a model that, however, requires direct experimental verification.

This study has several limitations. First, RNA-seq was performed using planktonic monocultures and may not fully represent transcriptional programs operating in mature biofilms or during interspecies interactions (Arbulu et al., 2025; Rose et al., 2023). Biofilm-resolved and dual-species transcriptomic analyses, with species-specific read assignment against both genomes, will be needed to directly link ZccR-controlled gene expression to the observed biofilm and competition phenotypes. Most transcriptional changes appear secondary to zinc accumulation rather than direct ZccR binding, and the promoter elements for ZccR-independent zinc repression of *zccR* remain uncharacterized. The proposed mechanistic hypotheses, including impaired mutacin maturation/activity, reduced oxidative-stress tolerance, and altered thiol/glutathione metabolism, are based on transcriptomic associations and require direct validation at the protein, metabolite, and functional levels. These experiments represent important directions for future research.

Zinc is ubiquitous in the human diet and is widely incorporated into oral care and therapeutic products because of its antimicrobial properties (Uwitonze et al., 2020). Compared with other oral resident bacteria, *S. mutans* exhibits significantly higher zinc tolerance, which allows it to maintain a competitive growth advantage under this environmental factor (Ganguly et al., 2022). This study demonstrates that disrupting the ZccR-mediated zinc homeostasis system inhibits the growth of *S. mutans* in a zinc-enriched environment and reverses this tolerance advantage, rendering *S. mutans* less competitive in *S. mutans-S. gordonii* dual-species biofilms. In principle, interfering with ZccR function, together with the zinc exposure already present in the oral environment, could compromise the zinc tolerance of *S. mutans* and thereby shift the balance of dental plaque communities away from cariogenic species. However, this approach remains a possible future intervention point, and the ecological consequences of zinc exposure for the oral microbiome require careful evaluation.

## 5 Conclusion

ZccR is essential for zinc tolerance, biofilm formation, oxidative resistance, and the interspecies competitiveness of *S. mutans*. Its absence triggers widespread transcriptional remodeling. It activates the zinc efflux gene *zccE* and represses its own promoter. Zinc regulates *zccE* expression in a ZccR-dependent manner and inhibits *zccR* transcription through an independent pathway to maintain zinc homeostasis. Collectively, these findings indicate that ZccR links zinc homeostasis, biofilm formation, and bacterial interactions in *S. mutans*. Targeted modulation of the ZccR zinc efflux system to adjust biofilm composition may offer one approach for cariogenic biofilm control.

## Data Availability

The datasets presented in this study can be found in online repositories. The names of the repository/repositories and accession number(s) can be found below: https://www.ncbi.nlm.nih.gov/bioproject/PRJNA1493331, PRJNA1493331.

## Supplementary material

Supplementary Figure 1 Differential expression levels of genes *gtfB*, *gtfC*, and *gtfD* in *S. mutans* strains treated with 150 μM Zn^2+^. Gene expression levels were normalized to 16S rRNA gene transcript levels and are presented as mean ± SD. Group differences for each gene were evaluated using one-way ANOVA followed by Holm-corrected pairwise t-tests.

Supplementary Figure 2 Inhibitory effect of *S. mutans* strains against *S. salivarius* and *E. faecalis* under 150 μM Zn^2+^ treatment. Cells of *S. mutans* strains (5 μL) were inoculated onto half-strength BHI agar plates containing 150 μM Zn^2+^ and incubated for 24 h. Overnight cultures of *S. salivarius* and *E. faecalis* were subsequently diluted in 0.75% (w/v) soft agar and overlaid onto the plates. The plates were incubated at 37 °C under 5% CO_2_ for a further 24 h before imaging.

Supplementary Figure 3 RT-qPCR validation of differentially expressed genes identified by RNA-seq in the *ΔzccR* mutant versus the wild-type strain UA159 under 150 μM Zn^2+^ stress. Panels are grouped by functional category: (A) *zccE* (ZccR direct target). (B) Mutacin biosynthesis and transport genes *nlmA*, *nlmB*, *nlmC*, *nlmD*, *nlmE*, and *nlmT*. (C) Iron-uptake (*smu.995–smu.998*) and mutanobactin genes (*mubA*, *mubX*); (D) sulfur/cystine-uptake and nitrogen-metabolism related genes (*homR*, *tcyD–tcyH*, *glnA*, *glnB*, and *nrg*). (E) Carbohydrate-transport and metabolism genes (*lacA–lacG*, *lacR*, *scrA*, and *msmX*). Data indicate the mean ± standard deviation for three replicates. Relative gene expression was quantified using the 2^–ΔΔ Ct^ method. Two-sample t-tests with Holm correction were utilized to compare each gene between the Δ*zccR* mutant and wild-type UA159 strains. (**P* < 0.05; ***P* < 0.01; ****P* < 0.001)

Supplementary Table 1 Bacterial strains, plasmids, and primers used in this study.

Supplementary Table 2 List of genes differentially expressed greater than 2.0-fold between the wild type and Δ*zccR* strain cultured with 150 μM Zn^2+^.

Supplementary Table 3 List of genes expressed in the wild-type and Δ*zccR s*train cultured with 150 μM Zn^2+^.

Supplementary Table 4 List of genes expressed inΔ*zccR* strain cultured with versus without 150 μM Zn^2+^.
